# Chemoprotective effects of butanol fraction *of Buchholzia coriacea* (Capparidaceae) against type 2 diabetes and oxidative stress in male Wistar rats

**DOI:** 10.1042/BSR20170665

**Published:** 2019-02-15

**Authors:** Amanda C. Okolie, Oluwafemi E. Kale, Odutola Osilesi

**Affiliations:** 1 Department of Biochemistry, Benjamin S Carson (Snr.) School of Medicine, Babcock University, Ilisan-Remo, Ogun State, Ikeja 21244, Nigeria; 2 Department of Pharmacology, Benjamin Carson (Snr.) School of Medicine, Babcock University, Ilisan-Remo, Ogun State, Ikeja 21244, Nigeria

**Keywords:** Buchholzia Coriacea, Glibenclamide, Insulin Resistance, Oxidative Stress, Type 2 diabetes, β cells dysfunction

## Abstract

Recent studies have shown that Type 2 diabetes (T2D) in rats can result through a synergy that links obesity to insulin resistance and β-cell dysfunction. The present study achieved T2D via high fructose (20%^w/v^, p.o.), streptozotocin single dose (40 mg/kg, i.p.) (HFSTZ) in rats. Also, chemoprotective potential of butanol fraction of *Buchholzia coriacea* (BFBC) was demonstrated. Control normal and diabetic untreated (HFSTZ-induced T2D) rats received CM-cellulose (1 mg/kg, p.o.). Diabetic rats received intragastric BFBC (20, 200, 400 mg/kg), glibenclamide (0.07 mg/kg), and BFBC (200 mg/kg) plus glibenclamide treatments, respectively. 2,2-Diphenyl-1-picrylhydrazyl, nitric oxide radical, hydroxyl radical scavenging activities, and α-amylase inhibition were assessed. After 2 weeks of treatments, blood glucose levels, lipid profiles, renal and liver function, serum insulin as well as *in vivo* oxidative stress biomarkers were assessed. BFBC shows highest antioxidants and α-amylase inhibitory activities *in vitro*. HFSTZ-induced T2D produced hyperglycemia (*P*<0.05–0.001; F = 5.26–26.47), serum hyperinsulinemia (six-folds) plus elevated lipid peroxidation levels. Similarly, there were altered lipid profiles, liver and renal biomarker enzymes plus weight loss. BFBC administration alone or in combination with glibenclamide reversed T2D symptomatologies in treated animals, and improved body weights against control diabetic rats. *In vivo* antioxidant activities also improved while histological sections in treated rats show reduced tissue damage in pancreas, kidneys, liver, and heart, respectively. Oleic, stearic, 2-methyl-pyrrolidine-2-carboxylic, and n-hexadecanoic acids were present in BFBC in large quantities given GC-MS analysis. Overall, data from the present study suggest chemoprotective potentials of BFBC against HFSTZ-induced T2D rats.

## Introduction

Type 2 diabetes (T2D), a heterogeneous disorder, is a single term for a progressive decline in insulin action (insulin resistance (IR)) followed by the inability of pancreatic β-cells to compensate for IR (pancreatic β-cells dysfunction) [1]. Streptozotocin (STZ) is a prototype diabetogenic agent known to directly target pancreatic β-cells to stop insulin production. Its effect on the β-cells is well understood [2]. However, in rat experimental models, there have been discrepancies over the sole diabetes achieved via STZ administration [3]. First, a very high dose is required. Also, type 1 than type 2 symptoms are prevalent. More so, fructose feeding (FF) has been known to induce IR [4] and hyperinsulinemia in normal rats [5]. This has been used to explain the classical pathways of obesity-associated mechanisms of fructose ingestion and satiety response or an independence of weight gain in energy intake that characterized fructose-induced IR [6]. Although, STZ has been used extensively in the development of diabetes [7,8], however, evidences abound that it is unable to induce IR directly but rather results in hyperglycemia from direct pancreatic β-cell damage. On the other hand, FF has been supplied *ad libitum* either in drinking water or compounded in diets with a minimal concentration for a short or longer period to induce IR or T2D respectively, in rodents [4,5]. In T2D mellitus (T2DM) and other metabolic syndromes, oxidative stress is increased, although, this appears to underlie the development of cardiovascular disease, T2DM and diabetic complications [9]. In addition, some metabolites of lipid peroxidation have been implicated and seem to increase in the patients with obesity, metabolic syndrome as well as T2DM [10]. Thus, with a view to ascertain the involvement of FF when combined with STZ in an experimental animal T2D, in this study, we assessed oxidative stress biomarkers in vital organs where metabolic disorders may thrive. Recent studies have now demonstrated that stable T2D in rats can be obtained through a synergy that links obesity to IR and T2D [11–13]. This may, in part, help to mimic the symptoms of human disease with great propensity toward sourcing for new antidiabetic agents.

The use of plants as herbal remedies against several diseases that constitute economic problems such as diabetes is fast gaining recognition and publicity in Africa [14,15] with claims that they are relatively cheap, affordable, effective, perceived low toxicity with absence or minimal side effects. Consequently, some medicinal plants have become relevant and have gained scientific acceptability as an efficacious treatment for diabetes mellitus and other ailments [16]. The estimation of the number of diabetic patients by World Health Organization (WHO) in 1999 was 140 million and this number is expected to have doubled in another 20 years if there are no drastic intervention measures [17].

*Buchholzia coriacea* (BC), commonly known as wonderful kola, is a perennial plant which grows as a tree. It belongs to the family Capparidaceae and genus *Buchholzia* [18]. Its use in folklore medicine is popular for diabetes. Evidences abound for its diverse medicinal potentials [19–25]. However, study to demonstrate its involvement in T2D experimental animal model is poorly reported. Also, the bioactive constituents present in BC have not been characterized.

Therefore, in the present study, first, we demonstrated the potentials of butanol fraction of BC (BFBC) in HFSTZ-induced T2D in male Wistar rats both *in vitro* and *in vivo* with a view to ascertain its chemoprotective benefits. Also, we identified the bioactive compounds present in BFBC using GC-MS.

## Methods

### Collection of plants

Fresh seeds of BC were obtained from a farm in Aku, Igbo-Etiti North Local Government Area of Nsukka in Enugu state, Nigeria. Some seeds were deposited, identified, and authenticated by G.A. Ademoriyo, a botanist, at the herbarium of the Obafemi Awolowo University, Ile-Ife, Nigeria. A voucher specimen assigned reference number IFE-17574 was deposited in the institutional herbarium.

### Extractions

Properly rinsed seeds of BC were air-dried at 24 ± 1°C for 2 weeks and pulverized mechanically using a miller grinder. A known weight of the dried sample was soaked in 70% methanol using the ratio 1:8^w/v^. After 48 h, the filtrate obtained was concentrated in a rotary evaporator at 39 ± 1°C and percentage yield estimated. The concentrate obtained was reconstituted in distilled water and partitioned with hexane, ethylacetate, and butanol, respectively. The various solvent fractions were further concentrated and stored at 4°C prior to analysis. The percentage weight yields of different fractions of butanol, ethyl acetate, and hexane were compared with methanolic seeds extract of BC. Percentage weight yield was calculated as: *n/N* × 100 (n, weight of concentrate, N, weight of BC powder or methanolic extract concentrate).

### Experimental animals

Adult male Wistar rats (230 ± 20 g) were obtained from the Banjamin Carson (Snr.) School of Medicine, Babcock University Laboratory Animal house, Ilishan Remo, Ogun state, Nigeria. They were housed in a unisexual group of four in a metallic cage (60 × 45 × 25 cm) under a reversed light–dark cycle (12 h/12 h dark scheduled) and controlled temperature (22 ± 3°C). The animals were acclimatized for 2 weeks. They were fed with commercially available pelleted diet (Vita Feeds, Jos, Plateau State, Nigeria) and water *ad libitum* during the period of acclimatization and throughout the period of the experiment. The study was carried out in compliance with established guidelines for biomedical research as approved by the Babcock University, Ogun State, Nigeria in conjunction with the organization for Animal Care and Use in Research, Education and Testing (ACURET.ORG). The investigation conforms to the Guide for the Care and Use of Laboratory Animals published by the U.S. National Institutes of Health (NIH Publication No. 85-23, revised 1996) for studies involving experimental animals and the procedures as documented by Kilkenny et al. [26] for reporting animal research. An ethical clearance was obtained from the Babcock University Human Research Ethics Committee (BUHREC 489/16).

### Drugs and chemicals

Glibenclamide was obtained from Pharmacare Ltd., Durban, South Africa. STZ (Sigma–Aldrich), Fructose (Burgoyne Reagents, India), α-Amylase (Sigma–Aldrich), Acarbose-Glucobay (Bayer Pharma, India), Reduced glutathione, metaphosphoric acid, and trichloroacetic acid were purchased from J.I. Baker (Center Valley, PA, U.S.A.). Thiobarbituric acid was purchased from Sigma Chemical Company (U.S.A.). Alanine aminotransferase (ALT), aspartate aminotransferase (AST), alkaline phosphatase (ALP), total cholesterol, (TC) and triglyceride (TG) assay kits were obtained from Randox Laboratory (Crumlin, U.K.), 5′,5′-dithiobis-2-nitrobenzoate (Ellman’s reagent) from Sigma (U.S.A.) and sodium hydroxide from Merck (Germany). Rat insulin enzyme-linked immunoassay kit (RAB0904, Rat Ins1/Insulin ELISA) was purchased from Sigma–Aldrich (U.S.A.). Other chemicals and reagents used were of analytical grade.

### Determination of total phenolic content and flavonoid

The total phenol and tannin content was determined by the Folin–Ciocalteu procedure by Skerget et al. [27]. The total flavonoid content was determined by the aluminium chloride calorimetric assay [28].

### *In vitro* antioxidant assays

The free radical scavenging activity for 2,2-Diphenyl-1-picrylhydrazyl (DPPH) assay, was measured as described previously by Manzocco et al. [29]. The nitric oxide (NOS) and hydroxyl radical scavenging (HRS) activities were assessed by the methods described by Marcocci et al. [30] and Halliwell et al. [31], respectively.

### *In vitro* α-amylase inhibition study

Briefly, the activity of α-amylase was measured using the starch-iodine method described by Komaki et al. [32] with slight modifications. A 20 μl α-amylase solution (0.030 mg/ml) was mixed with 1.3 ml of phosphate buffer (0.02 M containing 0.0067 M NaCl, pH 6.9) and various concentrations of the fractions (20–100 μl). After incubation at 37°C for 20 min, 100 μl of 0.1% starch solution was added, and the mixture re-incubated for 20 min, after which 2 ml of 0.01% acidic iodine solution was added and the absorbance measured at 565 nm. The percentage inhibition was calculated for each sample relative to control. The α**-**amylase inhibition of enzyme activity was estimated as reported by Etokakpan and Palmer [33].

### Acute oral toxicity test

Acute oral toxicity test was performed using the test procedure as per organization for economic co-operation and development (OECD) guidelines 423 (OECD, 2001). Eighteen male Wistar rats (average weight: 117 g) were randomly divided into six groups each containing three rats and allowed to acclimatize for 2 weeks. Graded doses of BC extract of 200, 400, 1000, 2000, 3000, and 4000 mg/kg body weight were administered to the animals orally and were allowed access to water *ad libitum*. Control group received 0.2 ml distilled water orally. The animals were observed individually for at least once during first 30 min after dosing, periodically for 24 h and daily post-treatment for mortality. Behavioral changes including hyperactivity, irregular movement, leaning on hind limbs, hyperphagia, scratching of lower jaw immediately after treatment and 2 h after treatment, writings, respiratory abnormality and agitation were observed. All the animals were further monitored for 14 days post administration.

### Experimental T2D induction

Fructose (20%^w/v^) solution was supplied to rats *ad libitum* for a period of 2 weeks. Animals were then supplied with normal drinking water during the remaining period of the experiment while the control group received distilled water uninterruptedly. Also, a single dose of STZ (40 mg/kg) [13] in citrate buffer (pH 4.4) was administered intraperitoneally (i.p.) on the 15th day of the experiment to all fructose-fed rats, whereas, the control normal group received vehicle citrate buffer (i.p.). One week following the STZ injection, fasting blood glucose levels (FBGLs) were measured using a portable Accu-check glucometer (Gluco-Plus Inc., Quebec, Canada) in the blood collected via the tail vein. Animals with FBGL > 260 mg/dl were considered as diabetic. The weekly FBGLs of all animals was measured similarly on days 4, 7, 10, and 14, respectively.

### Experimental design

Forty-two male Wistar rats were divided into seven of six rats/group. Treatments with an antidiabetic drug, glibenclamide (therapeutic dose: 0.07 mg/kg/day) and BFBC extracts commenced immediately when animals were classified into non-diabetic and diabetic groups. Group 1 (normal control) received oral 0.5% carboxymethyl cellulose (CMC) (1 ml/kg p.o.) throughout the experiment. Group 2 (control diabetic untreated, HFSTZ) received fructose (20%^w/v^, p.o. for 2 weeks) and single-dose STZ (40 mg/kg, i.p.) and then maintained on CMC (1 ml/kg, p.o.) during the remaining periods of the experiment, group 3 received glibenclamide (GLIB) (0.07 mg/kg/day, p.o.) while groups 4, 5, and 6 were administered 20, 200, and 400 mg/kg body weight of BFBC, respectively via oral gavage. In addition, group 7 received BFBC (200 mg/kg, p.o.) plus GLIB (0.07 mg/kg, p.o.), respectively. Diabetic animals were treated for 2 weeks and rats were killed 24 h following the last treatment. Animals’ changes in body weight, daily food, and fluid intake for normal, diabetic, and treated rats were measured and recorded per day.

### Necropsy

The animals were killed by cervical dislocation 24 h after the last treatment. Blood samples were collected by cardiac puncture into plain bottles and centrifuged at 4200 ***g*** (5 min) to separate serum. The liver, pancreas, kidneys, and heart were removed, cleared of adhering tissues, and placed immediately on 0.25 M ice-cold sucrose and weighed. The weight was recorded in grams and expressed as g/kg body weight. A small portion of each organ was carefully excised, fixed in 10% formaldehyde, dehydrated in graded alcohol, and embedded in paraffin. Fine sections were obtained, mounted on glass slides and counterstained with Hematoxyllin and Eosin (H&E) for histopathologic examination. The remaining portions were weighed and homogenized in four volumes of Tris/HCl buffer (0.1 M, pH 7.4). Both serum and homogenates were used for biochemical analysis.

### GC-MS analysis

GC-MS analysis of BFBC from seeds of BC was carried out using an Agilent HP- 7890A gas chromatograph (Agilent Technologies, Palo Alto, CA, U.S.A.) with HP-5MS 5% phenylmethylsiloxane capillary column (30 m × 0.25 mm, 0.25 lm film thickness; Restek, Bellefonte, PA) equipped with an MSD detector and characterized as previously described Proestos et al. [34] with some modifications. Oven temperature was maintained at 40°C for 3 min initially, and then raised at the rate of 3°C/min to 250°C. Injector and detector temperatures were set at 220 and 290°C, respectively. Helium was used as carrier gas at a flow rate of 1 ml/min, and diluted samples (1/1000 in n-pentane, v/v) of 1.0 µl were injected manually in the splitless mode. Peak area percents were used for obtaining quantitative data. In addition, GC-MS of BFBC was carried out on an Agilent HP 7890A gas chromatogram (Technology model MSD = 5975C detector) Agilent Technologies, Injector: 7683B Series, Ionization energy: 70 eV, Palo Alto, CA, U.S.A.). Initial temperature = 100°C held for 2 min, final temperature = 270°C at the rate of 10°C/min. One microliter of various fractions of the extract of the BFBC was injected. Temperature of heater was set at 250°C, pressure 3.2652 psi, mode type slit-less, column type (HP 5MS: 30 m × 320 µm × 0.25 µm) and carrier gas (Helium, 99.9999% purity, flow rate = 1.4963 ml/min; average velocity = 45.618 cm/s). The constituent compounds were determined by comparing their retention times and mass weights with those of authentic samples obtained by GC as well as the mass spectra. GC-MS interpretation on mass spectrum was conducted using the database of National Institute Standard and Technology (NIST) version 2.0 MS. NIST database has more than 62000 patterns. The spectrum of the unknown component was compared with the spectrum of the known components stored in the NIST library.

### Assessment of hepatic function

Serum AST, ALT, and ALP activities were assessed for liver function. AST and ALT activities were determined according to the principle described by Reitman and Frankel [35] while the ALP activity was carried out according to the method described by Roy [36]. To assess the synthetic function of the liver, kidneys, pancreas, and heart, total protein concentration was carried out according to the principle based on Biuret reaction [37].

### Antioxidants assessment

#### GSH, GPx, SOD, CAT determination and lipid peroxidation assay

GSH level was estimated at 412 nm following the method of Beutler et al. [38]. Lipid peroxidation level was estimated spectrophotometrically by the thiobarbituric acid-reactive substance (TBARS) using the method as described by Varshney and Kale [39] and expressed in terms of malondialdehyde (MDA) formed per mg protein. Superoxide dismutase (SOD) and catalase activities were determined following the methods of Misra and Fridovich [40] and Sinha [41], respectively. Glutathione peroxidase (GPx) activity followed the method described by Ellerby and Bredesen [42].

#### Total cholesterol and triglyceride assay

Serum total cholesterol (TC) and triglyceride (TG) concentrations were estimated following the principle described by Trinder [43] using commercial kits obtained from Randox Laboratories Ltd (Crumlin, U.K.). Uric acid was also determined using Randox kit following the principle described by Fossati et al. [44]. The method described by Warnick and Albers [45] was used to determine high-density lipoprotein (HDL) while low-density lipoprotein (LDL) was extrapolated using Friedewald et al. [46] formula.

### Histological assessment

The pancreas, liver, heart, and kidney portions for histological examination were passed through the process of fixation, dehydration, clearing, infiltration, embedding, sectioning, and staining, although, only approximately 4–5 mm thickness was trimmed for proper fixation. All tissues were then fixed in 10% formol saline and were then transferred to 50% alcohol (60–100%), for 2 h. Alcohol was removed from the treated tissues by titrating them through first an equal mixture 100% (absolute) alcohol and xylene for 1 h each in that order. Infiltration was carried out twice by passing each tissue through molten paraffin wax in an oven at a temperature of 30°C for one and a half hours each. The tissues so embedded in molten paraffin wax were later placed on a wooden block and trimmed to size. Serial sections, 10-μm thick, were made using a rotatory microtome. The cut sections were then floated in a warm water bath at a temperature of 30.5–40.5°C and placed on slides. Approximately eight sections were obtained from each treated organ from each animal. Four samples were placed on each slide. Microscopic examination was done by using varying magnifications of 40, 100, and 400 to determine if the samples were properly fixed on the slides. Following staining, mounting of sections was carried out using dimethyl paraffinate xylene (DPX) as a mounting agent, after which microscopic examination was done.

### Measurement of serum insulin

An ELISA for rat insulin was followed using the method described previously by Webster et al. [47] with slight modifications. One hunred microliters of anti-target antibody was added to each well and incubated for 1.5 h at 25°C with gentle shaking. This was discarded and washed in wells four-times in 250 μl buffer each prior to inversion. One hunred microliters of each standard, positive control, and sample were added into appropriate wells, enclosed, and incubated for 2.5 h at 25°C with gentle shaking. This was discarded again and washed four-times as directed above. Afterward, 100 μl of prepared horseradish peroxidase–streptavidin solution was added to each well and then incubated for 45 min with gentle shaking at 25°C. Solution was discarded and washed four-times as aforementioned. Another 100 μl of 3′,3′,5′,5′-tetramethylbenzidine (TMB), one-step substrate reagent, was added to each well, and incubated for 30 min at 25°C in the dark with gentle shaking. Finally, 50 μl of stop solution was added to each well and the absorbance was read at 450 nm immediately according to the manufacturer’s instructions.

### Statistics

Results were expressed as mean ± S.E.M. Differences between groups were determined by one-way ANOVA and *t* test using Statistical Package for Social Sciences (SPSS, 20.0) software for windows. Post-hoc testing was performed for intergroup comparisons using the least significant difference (LSD), followed by Dunnett’s test [48]. Graphical presentations were achieved using GraphPad Prism 6 and the *P*-value <0.05 was considered significant.

## Results

The methanolic extract of BC yielded 29%. However, percentage yields for n-butanol fraction was highest followed by ethyl acetate and n-hexane of BC (11.3 > 5.38 > 3.26), respectively.

Acute oral toxicity testing using methanolic extract of BC showed no mortality following administration of graded doses of BC extracts of 200–4000 mg/kg body weight. Control group also received 0.2 ml distilled water orally. However, within the first 2 h of administration, animals administered 1000–4000 mg/kg exhibited behavioral abnormality which manifested as abdominal contraction, hyperactivity, leaning on hind limbs, writing drowsiness, and scratching of lower jaw r,espectively. All the animals were further monitored for 14 days post administration.

**Figure 1:**
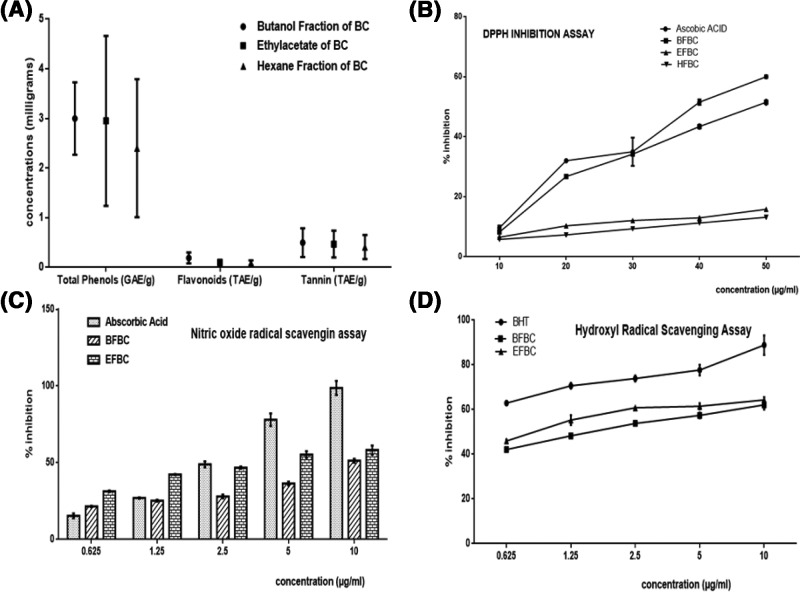
Shows phytochemical analyses of various fractions of BC seed extracts Results are represented as mean ± S.E.M.; *n*=3. (**A**) Quantitative phytochemical, (**B**) 1-1-Diphenyl 2-picryl hydrazyl (DPPH) activities, (**C**) nitric oxide radical scavenging (NORS) activities, and (**D**) hydroxyl radical scavenging activities (HRSA). Abbreviations: BHT, butylhydroxytoluene; EFBC, ethylacetate fraction of BC; g, gram; GAE, gallic acid equivalent; QE, quercetin equivalent; TAE, tannic acid equivalent.

Figure 1A: BFBC has the highest content of phenols, flavonoids, and tannins when compared with the EFBC or HFBC, although, EFBC contains more than HFBC.

Figure 1B: Different concentrations of extract fractions were compared with ascorbic acid standard (IC_50_ = 10–50 µg/ml). From the results obtained, BFBC demonstrated highest antioxidant activities than EFBC or HFBC at all the concentrations used (10–50 µg/ml) by 24.1, 16.5, 2.2, 15.7, and 14.2%, respectively against ascorbic acid standard. However, EFBC shows better antioxidant actions than HFBC.

Figure 1C: Different concentrations of extract fractions were compared with ascorbic acid standard (0.625–10 µg/ml). From the results obtained, EFBC and BFBC at 0.625 and 1.25 µg/ml, increased NOS activity by 123.5, 56.8, and 39.6%, respectively. However, BFBC showed low antioxidant activity when compared with ascorbic acid standard. In contrast, however, both BFBC and EFBC decreased NOS activity at concentrations 2.5 µg/ml (42.7 and 4.4%), 5 µg/ml (53.8 and 29.1%), and 10 µg/ml (48 and 41%), respectively.

Figure 1D: Different concentrations of extract fractions were compared with ascorbic acid standard (0.625–10 µg/ml). From the results obtained, although, both fractions decreased HRS activity. BFBC showed better antioxidant activities than EFBC at 2.5, 5, and 10 µg/ml by 27.2, 26.1, and 30.1%, respectively when compared with control.

**Figure 2:**
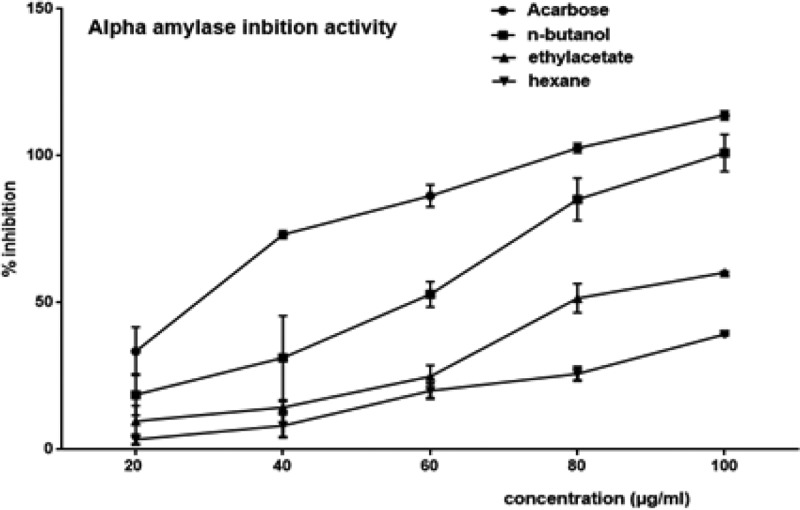
Inhibitory effects on α-amylase activities of various fractions of BC seed extracts Results are represented as mean ± S.E.M.; *n*=3. Abbreviations: EFBC, ethylacetate fraction of BC; HFBC, hexane fraction of BC*.*

Figure 2: Results show that BFBC demonstrated significant (*P*<0.05) α-amylase inhibitory activities at the concentrations used (20–100 µg/ml). Also, α-amylase inhibitory activities were higher in EFBC than HFBC, however, BFBC has highest percentage α-amylase inhibitory activity which is concentration dependent. Although, the α-amylase activity of the most potent fraction, BFBC, was lower than that of the standard when compared at low concentrations of the extracts between 20 and 60 µg/ml, the IC_50_ was slightly higher at the highest dose in acarbose. But, there were no significant difference (*P*>0.05) given the highest doses (80–100 µg/ml) of BFBC when compared with the standard acarbose.

**Figure 3:**
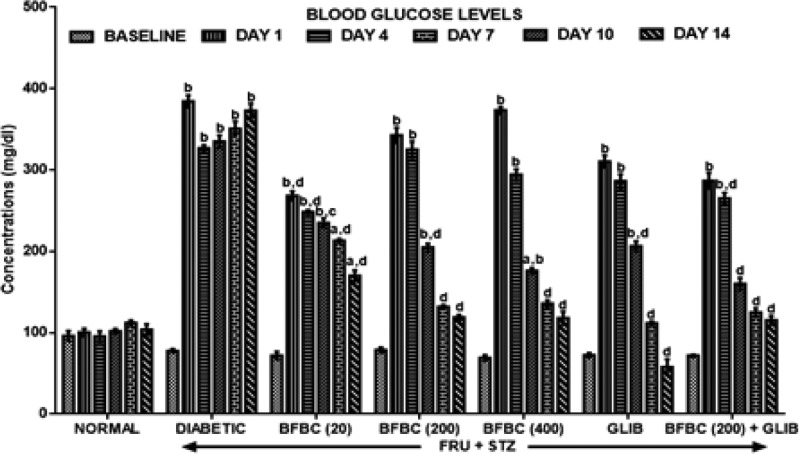
Effects of butanolic fraction of BC extract on FBGLs of normal and HFSTZ-induced T2D in male Wistar rats Results are represented as mean ± S.E.M.; *n*=6. ^a^*P*<0.05 or ^b^*P*<0.001 compared with control normal group. ^c^*P*<0.05 or ^d^*P*<0.001 compared with control diabetic group. Abbreviation: HFSTZ, high-fructose STZ.

Figure 3: On day 1 (following HFSTZ administration), the diabetic untreated group showed significantly elevated fasting blood glucose levels (FBGL) by at least three-folds (394.6%) when compared with control normal baseline group. But, after 2 weeks of treatment (day 14), the FBGL in the HFSTZ-induced T2D untreated rats remained elevated (*P*<0.05) when compared with the baseline. However, an antidiabetic drug, GLIB, was able to lower FBGL by day 4 (296.5%), day 7 (185.7%), day 10 (54.7%), and by day 14 (20.1%), respectively. All doses of BFBC show reduced (*P*<0.0.5–0.001) FBGL in a time-dependent manner as follows: 20 mg/kg; day 4 (88.5%), day 7 (226.4), day 10 (195.8%), and day 14 (136.1%), 200 mg/kg; day 4 (312.9%), day 7 (160.1%), day 10 (67.3%), day 14 (50.8%), and 400 mg/kg; day 4 (326.5%), day 7 (155.1%), day 10 (96.7%), and day 14 (71.1%), respectively. Also, BFBC + GLIB combination produced decreased (*P*<0.05) FBGL in a time-dependent manner by 7.6% (day 4), 44.2% (day 7), 56.5% (day 10), and 59.8% (day 14), respectively.

**Figure 4:**
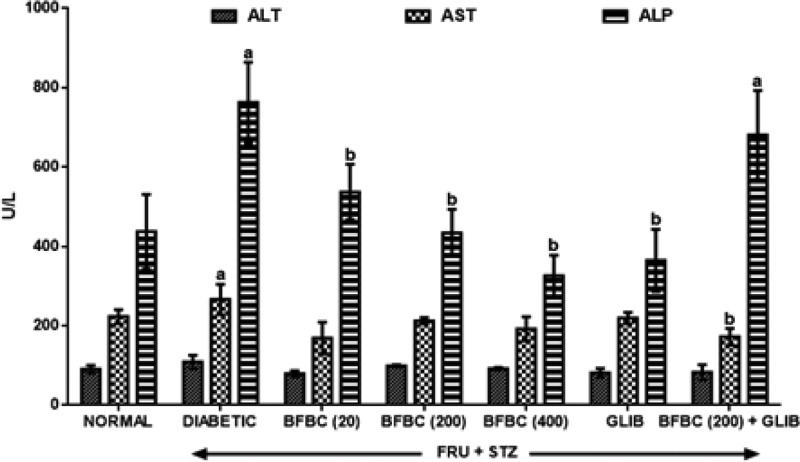
Effects of butanolic fraction of BC extract on serum ALT, AST, and ALP levels of normal and HFSTZ-induced T2D in male Wistar rats Results are represented as mean ± S.E.M.; *n*=6. ^a^*P*<0.05 when compared with control normal group, ^b^*P*<0.05 when compared with control diabetic group. Abbreviation: HFSTZ, high-fructose STZ.

Figure 4: HFSTZ-induced T2D rats show elevated serum ALT (*P*<0.05, 20%), AST (*P*>0.05, 19%), and ALP (*P*<0.05, 74%), respectively when compared with control normal group. BFBC reduces ALT, AST, and ALP levels by 27.5, 36.6, and 25.7% (20 mg/kg), although, in a non-dose-dependent manner when compared with control diabetic group. However, similarly, a dose-dependent reduction was achieved in ALT, AST, and ALP levels at 9.2, 20.3, 43.1% (200 mg/kg) and 15.6, 27.8, 57.2% (400 mg/kg), respectively. In addition, GLIB (0.71 mg/kg) treated rats show reduced levels of ALT, AST, and ALP in treated rats by 25.4, 17.3, 52.1%, respectively when compared with control diabetic group.

**Figure 5:**
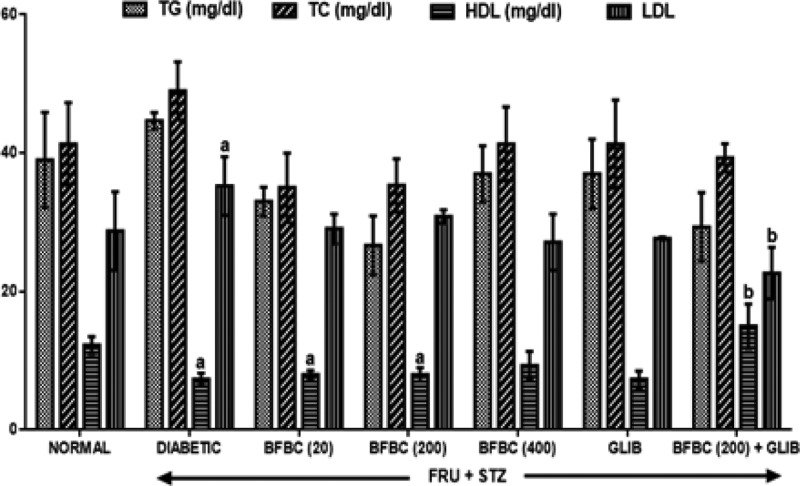
Effects of butanolic fraction of *b. coriacea* extract on the lipid profiles levels of normal and HFSTZ-induced T2D in male Wistar rats Results are represented as mean ± S.E.M.; *n*=6. ^a^*P*< 0.05 when compared with control normal group, ^b^*P*<0.05 when compared with control diabetic group. Abbreviation: HFSTZ, high-fructose STZ.

Figure 5: From the results obtained, HFSTZ-induced T2D untreated rats show increased (*P*>0.05) serum TC and TG levels when compared with control normal group. Similarly, LDL level increased (*P*<0.05) in the diabetic rats. Also, HDL was reduced (*P*<0.05) in HFSTZ-induced T2D diabetic rats group when compared with control normal group. BFBC when administered at doses of 20, 200, and 400 mg/kg reduced (*P*>0.05) TG (40, 17, 17%) and TC (28, 16, 16%) when compared with control diabetic animals. BFBC doses of 20 and 400 mg/kg increased (*P*>0.05) HDL by 9 and 27%, respectively. Further, an administration of BFBC of 20, 200, and 400 mg/kg reduced the elevated LDL in a dose-dependent manner by 13, 22, and 23%, respectively when compared with control diabetic untreated group.

**Figure 6:**
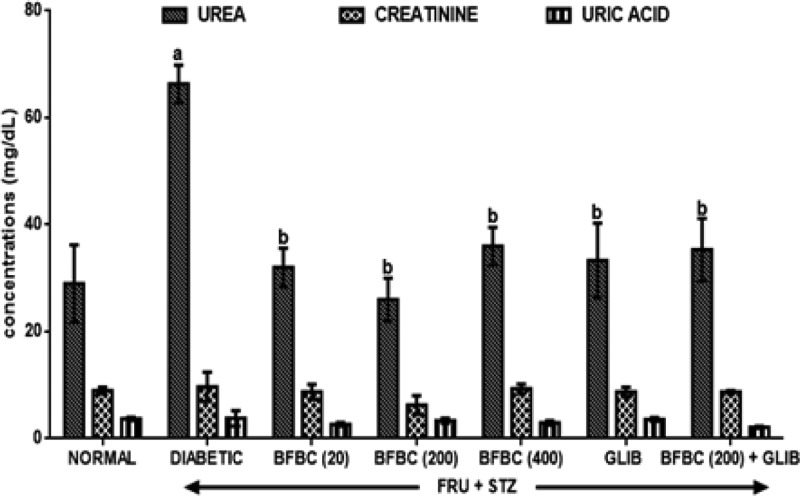
Effects of butanolic fraction of BC extract on serum urea, creatinine, and uric acid levels of normal and HFSTZ-induced T2D in male Wistar rats Results are represented as mean ± S.E.M.; *n*=6. ^a^*P*<0.05 or ^b^*P*<0.001 when compared with control normal group. ^c^*P*<0.05 when compared with control diabetic group. Abbreviation: HFSTZ, high-fructose STZ.

Figure 6: HFSTZ-induced T2D rats increased serum urea (*P*<0.05, 128.6%), creatinine (*P*>0.05, 7.8%), and uric acid (*P*>0.05, 2.7%), respectively, when compared with control normal group. GLIB also reduced (*P*<0.05) urea levels by 52% respectively when compared with control diabetic group. It also reduced (*P*>0.05) creatinine (10%) and uric acid (30%) levels in treated animals. BFBC in treated rats significantly reduced urea (*P*<0.05), 61% (20 mg/kg), 50% (200 mg/kg), and 46% (400 mg/kg).

Table 1: Diabetic rats show increased (*P*<0.05) in PCV and neurtrophil levels when compared with control normal group. Similarly, GLIB also increased (*P*>0.05) platelet and lymphocytes levels, respectively. More so, PCV was increased in BFBC treated rats that were administered 20 mg/kg (21.5%, *P*<0.05) and 400 mg/kg (13.8%, *P*>0.05) respectively when compared with control diabetic group. On the other hand, 200 mg/kg of BFBC increased (*P*<0.05) the PCV when compared with control normal group. HFSTZ-induced T2D rats and BFBC (400 mg/kg) lowered (*P*<0.05) by 64 and 49.4% lymphocyte counts compared with control normal group. However, lymphocyte count was elevated following an administration of 20, 200 mg/kg, and GLIB when compared with control diabetic group. More so, 200 mg/kg treated rats had reduced (*P*<0.05) neutrophils by 44.4%. Total white blood cells counts, hemoglobin and platelet levels were unaltered in all animals throughout the experiment.

**Table 1:** Shows the effects of butanolic fraction of BC extract on hematological indices of normal and fructose-fed, STZ-induced in male Wistar rats

GROUPS	PCV	HB	WBC	PLT	NEUT	LYM
Control	32.5 ± 0.5	10.8 ± 0.2	5.7 ± 1.5	7 ± 1.0	18.0 ± 1.0	82.0 ± 1.0
HFSTZ	40.5 ± 2.5^2^	13.40 ± 0.8	7.50 ± 0.7	14 ± 6.0	47.5 ± 1.5^2^	52.5 ± 1.5^2^
GLIB (0.07)	34.0 ± 1.0	11.25 ± 0.4	8.55 ± 0.9	15 ± 9.0	25.5 ± 2.5	74.5 ± 2.5
HFSTZ +						
BFBC (20)	39.5 ± 2.5^2^	14.75 ± 2.5	7.25 ± 1.2	20 ± 4.0	31.0 ± 0.9	68.0 ± 0.8
BFBC (200)^1^	31.0 ± 1.0	10.25 ± 0.4	4.25 ± 0.2	20 ± 2.0	10.0 ± 7.0^3^	89.5 ± 6.5
BFBC (400)	37.0 ± 1.0	12.25 ± 0.4	7.75 ± 1.9	15 ± 1.0	52.0 ± 4.0	41.5 ± 2.5^2^
GLIB + BFBC^1^	39.0 ± 3.0	12.35 ± 0.5	4.13 ± 3.3	36.5 ± 1.5	19.5 ± 5.5	80.0 ± 1.0

Results are represented as mean ± S.E.M.; *n*=6. Abbreviations: GRAN, granulocyte; HCT, hematocrit; HFSTZ, high-fructose STZ; HGB, hemoglobin; LYM, lymphocyte; NEUT, neutrophil; PCV, packed cell volume (%); PLT, platelet (× 10^4^/l); WBC, white blood cell (× 10^4^/l). ^1^Indicates 200 mg/kg. ^2^*P*<0.05 when compared with control normal group. ^3^*P*<0.05 when compared with control HFSTZ group.

Table 2: Untreated diabetic control rats show reduced (*P*>0.05) sodium, calcium, chloride ions, and bicarbonate levels, respectively when compared with control normal group. Conversely, a slight but an increased potassium ion level (6.5%, *P*>0.05) was obtained in HFSTZ-induced T2D rats. Also, GLIB produces slightly increased sodium (2.9 and 0.2%) and chloride levels (3%), respectively. BFBC when administered at 20, 200, and 400 mg/kg or in combination with GLIB did not significantly alter body electrolytes (bicarbonate, sodium ion, chloride ion, calcium ion, and potassium ion) levels when compared with control groups.

**Table 2:** Shows the effects of butanolic fraction of BC extract on blood electrolytes levels of normal and fructose-fed, STZ-induced in male Wistar rats

Treatment	HCO^3+^	Na^+^	K^+^	CI^−^	Ca^2+^
Control	23.0 ± 1.16	141.3 ± 2.73	4.6 ± 0.18	105.0 ± 1.15	8.9 ± 0.87
HFSTZ	24.7 ± 1.45	139.3 ± 1.20	4.9 ± 0.18	101.7 ± 2.96	8.6 ± 0.75
HFSTZ +					
BFBC (20)	21.3 ± 1.86	140.3 ± 0.88	4.4 ± 0.44	102.7 ± 0.88	8.8 ± 0.38
BFBC (200)	21.7 ± 0.67	144.3 ± 1.53	4.8 ± 0.12	104.0 ± 0.58	9.3 ± 0.29
BFBC (400)	20.7 ± 1.76	144.0 ± 2.00	5.0 ± 0.09	104.7 ± 1.33	9.5 ± 0.31
GLIB (0.07)	22.0 ± 0.58	139.7 ± 2.33	4.6 ± 0.47	104.7 ± 2.33	8.6 ± 0.93
GLIB+ BFBC (200)	18.7 ± 1.45	143.3 ± 1.86	5.0 ± 0.24	106.00 ± 1.00	8.8 ± 0.03

Results are represented as mean ± S.E.M.; *n*=6. Abbreviations: Ca, calcium ion; Cl, chloride ion; HCO, bicarbonate ion; HFSTZ, high-fructose STZ; K, potassium ion; Na, sodium ion.

**Figure 7:**
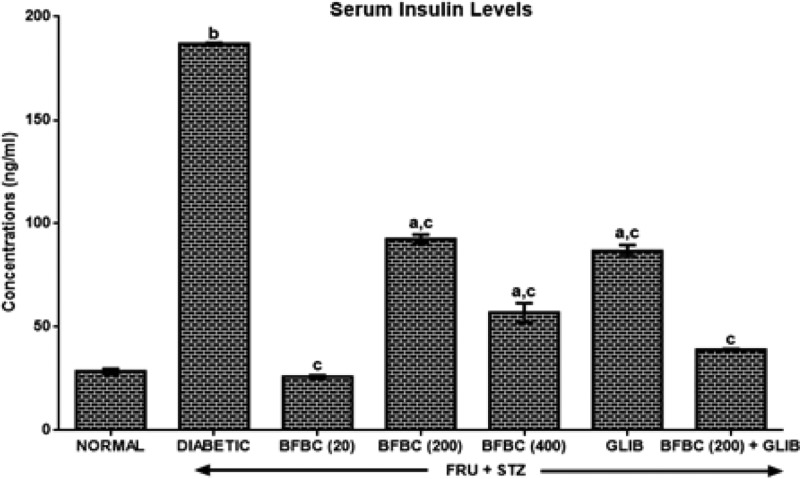
Effects of butanolic fraction of BC extract on serum insulin levels of normal and HFSTZ-induced T2D in male Wistar rats Results are represented as mean ± S.E.M.; *n*=6. ^a^*P*<0.05 or ^b^*P*<0.001 when compared with control normal group. ^c^*P*<0.05 when compared with control diabetic group.

Figure 7: HFSTZ-induced T2D produces six-folds increase in serum insulin level by 556% when compared with control normal group. GLIB, however, lowered insulin levels by 86% compared with control diabetic group while BFBC produces a dose-independent decrease (*P*<0.05) in serum insulin levels by 86.2% (20 mg/kg), 50.5% (200 mg/kg), and 69.6% (400 mg/kg), respectively.

**Figure 8:**
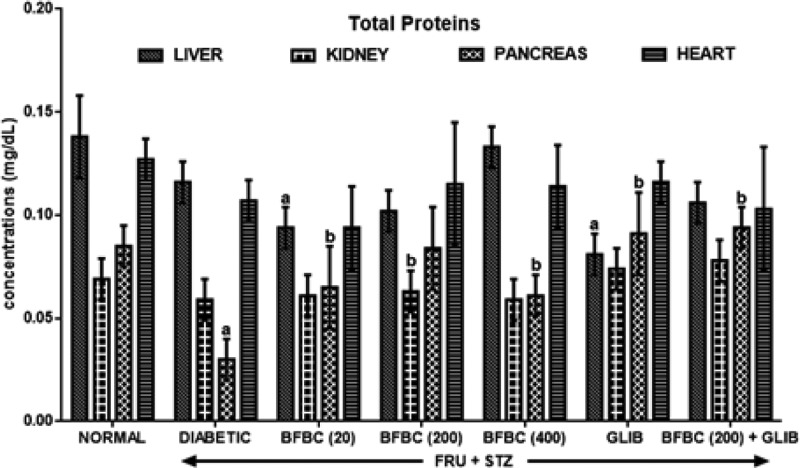
Effects of butanolic fraction of BC on liver, kidney, pancreas, and heart total protein levels of normal and HFSTZ-induced T2D in male Wistar rats Results are represented as mean ± S.E.M.; *n*=6. ^a^*P*<0.05 when compared with normal control group. ^b^*P*<0.05 when compared with control diabetic group.

Figure 8: Diabetic untreated rats show significantly decreased (*P*< 0.05) protein levels in the pancreas by 65% when compared with control normal group. Similarly, protein levels were decreased (*P*>0.05) in the liver (16%), kidneys (15%), and heart (16%) when compared with control normal group. GLIB produces elevated protein levels in the pancreas and kidneys by 25, 203% (*P*<0.05), respectively. GLIB did not cause any significant change in liver and heart when compared with control diabetic rats, but it increased (*P*>0.05) protein level in the heart when compared with control normal rats. More so, BFBC increased protein levels significantly by 116% (*P*<0.05) in the pancreas and slightly in the kidney (*P*>0.05, 3%) when compared with control diabetic animals. At 200 mg/kg of BFBC, protein levels increased in the pancreas (*P*<0.05, 180%), kidney (*P*>0.05, 7%), and heart (*P*<0.05, 8%), respectively. Further, at the highest dose of the extract used (400 mg/kg), protein levels in the pancreas and heart were elevated by 103% (*P*<0.05) and 7% (*P*>0.05), respectively. BFBC, at all administered doses did not significantly alter protein levels when compared with control normal group.

**Figure 9:**
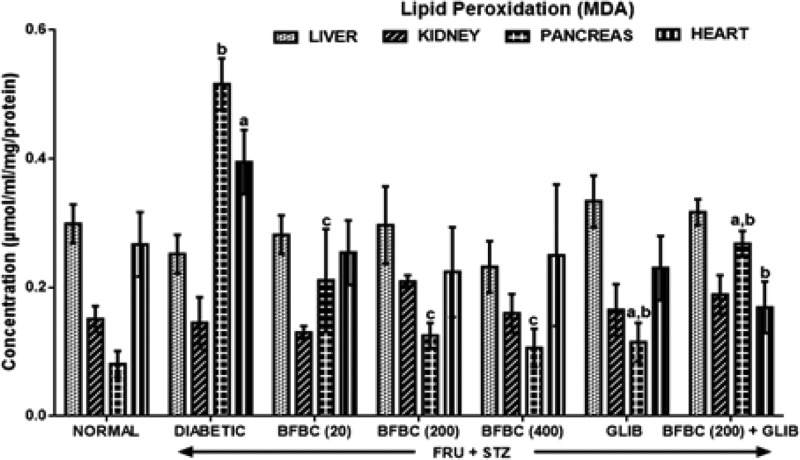
Effects of butanolic fraction of BC on liver, kidney, pancreas, and heart lipid peroxidation levels of normal and HFSTZ-induced T2D in male Wistar rats Results are represented as mean ± S.E.M.; *n*=6. ^a^*P*<0.05 or ^b^*P*<0.001 when compared with control normal group. ^c^*P*<0.05 when compared with control diabetic group.

Figure 9: Control diabetic animals show increased (*P*<0.05) MDA level measured in the pancreas up to six-folds by 537% and in the heart (49%, *P*<0.05) when compared with normal rats. But, MDA level was unaltered in liver and kidney when compared with control normal group. In BFBC treated groups, MDA levels were reduced in the pancreas in a dose-dependent manner; 20 mg/kg (*P*<0.05, 76%), 200 mg/kg (*P*<0.05, 78%), 400 mg/kg (*P*<0.05, 80%) while in the heart: 20 mg/kg (*P*<0.05, 24%), 200 mg/kg (*P*<0.05, 22%), 400 mg/kg (*P*<0.05, 15%). BFBC did not alter increased MDA levels in the liver and kidneys in the treated rats. Also, GLIB slightly reduced MDA levels by 59.1% (*P*<0.05) and 35.7%, respectively, with control diabetic rats in both pancreas and heart, respectively.

**Figure 10:**
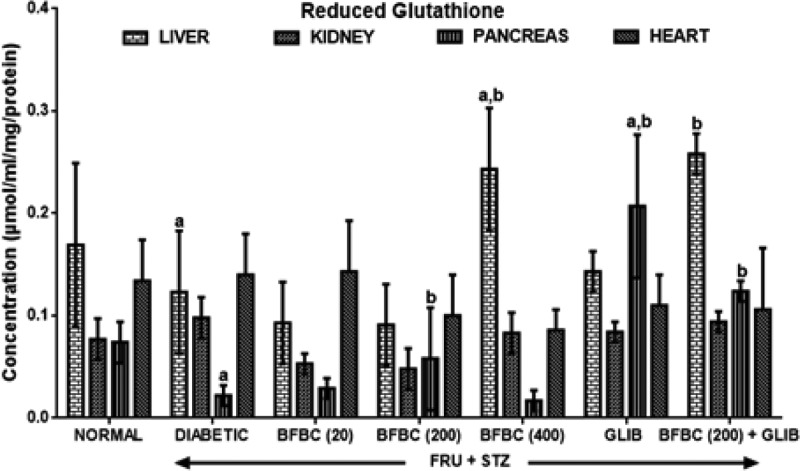
Effects of butanolic fraction of BC on liver, kidney, pancreas, and heart reduced glutathione of normal and HFSTZ-induced T2D in male Wistar rats Results are represented as mean ± S.E.M.; *n*=6. ^a^*P*<0.05 when compared with control normal group. ^b^*P*<0.05 when compared with control diabetic group.

Figure 10: Diabetic rats show significantly decreased (*P*<0.05) GSH levels by 70 and 27% in pancreas and liver, respectively, when compared with control normal group. GLIB causes increased hepatic and pancreatic GSH levels by 16 and 841% when compared with control diabetic group. More so, GLIB shows increased GSH levels in the kidneys (9%, *P*>0.05) and pancreas (180%, *P*<0.05) of treated rats when compared with control normal group. The BFBC at various doses demonstrated effectiveness that was not significantly different from that of GLIB in the present study. At lowest dose of BFBC (20 mg/kg) used, there was increased (*P*<0.05) in pancreatic GSH levels by 2.6-folds (163%) when compared with control diabetic rats. For the 200 mg/kg, GSH levels were elevated (*P*>0.05) in both the pancreas (32%) and heart (2%) when compared with control diabetic group. GSH levels were unaltered in the liver, kidneys, and pancreas in treated animals that received 200 mg/kg of BFBC compared with control normal group. However, an administration of 400 mg/kg produces elevated GSH levels in the liver (100%, *P*<0.05) when compared with control diabetic group.

**Figure 11:**
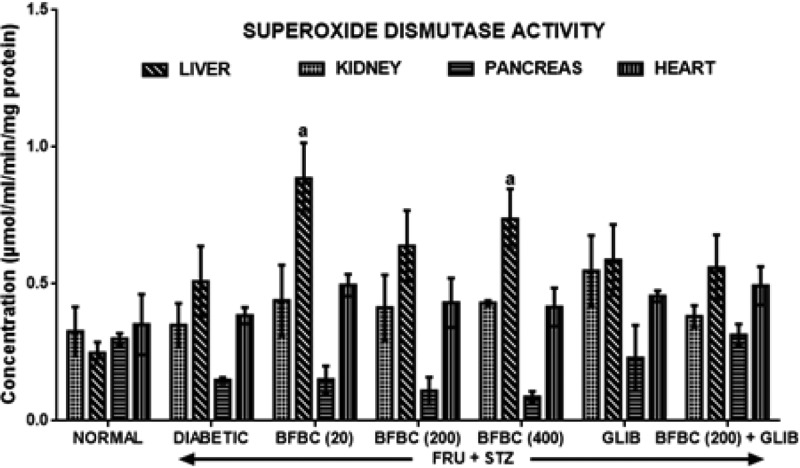
Effects of butanolic fraction of BC on liver, kidney, pancreas, and heart superoxide dismutase activities of normal and HFSTZ-induced T2D in male Wistar rats Results represented as mean ± S.E.M.; *n*=6. ^a^*P*<0.05 when compared with control normal group, ^b^*P*<0.05 when compared with control diabetic group.

Figure 11: Untreated diabetic rats produce increase in SOD activities in the liver, pancreas, and heart by 7% (*P*>0.05), 106% (*P*<0.05), and 9% (*P*>0.05), respectively, in control diabetic rats when compared with control normal group. Also, GLIB increased activities of SOD in liver (68, 57%, *P*>0.05), pancreas (183%, *P*<0.05) (16%, *P*>0.05), kidneys (nil) (102%, *P*<0.05), and heart (29, 19%, *P*>0.05) compared with control groups. Administration of BFBC (20 mg/kg) however increased SOD activities in the liver (26%, *P*>0.05), kidneys (74%, *P*<0.05), pancreas (1.4%, *P*>0.05), and heart (29%, *P*>0.05), respectively when compared with control diabetic group. Animals that received both 200 and 400 mg/kg produce similar increase in SOD activities in the liver (26, 45%), kidneys (26, 41%), and heart (12, 8%) but not in the pancreas. More so, BFBC (20, 200, 400 mg/kg) at the doses administered did not alter pancreas SOD activity.

**Figure 12:**
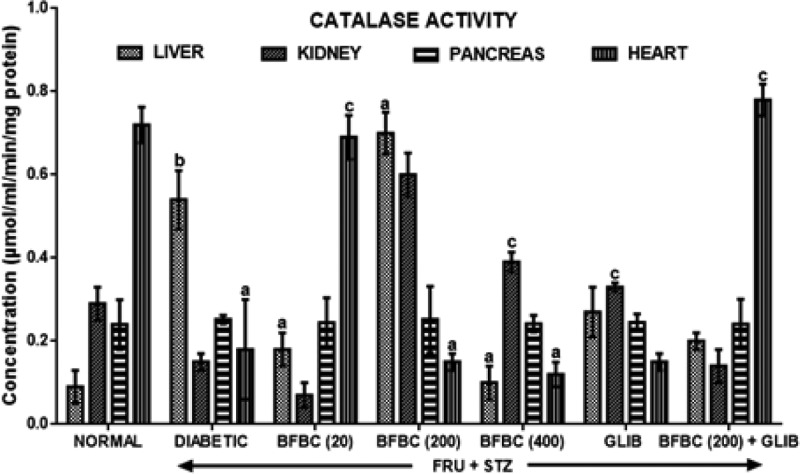
Effects of butanolic fraction of BC on liver, kidney, pancreas, and heart catalase activities of normal and HFSTZ-induced T2D in male Wistar rats Results are represented as mean ± S.E.M.; *n*=6. ^a^*P*<0.05 or ^b^*P*<0.001 when compared with control normal group. ^c^*P*<0.05 when compared with control diabetic group.

Figure 12: Diabetic rats show increased catalase activities in the liver and pancreas by 500% (*P*<0.001) and 5% (*P*>0.05), respectively, when compared with control normal group. Also, catalase activity decreased in the heart (*P*<0.05) when control diabetic animals were compared with control normal group. But, its activity in the kidneys was unaltered. GLIB produce increased kidneys catalase activity significantly (*P*<0.05) by 120%. Catalase activities were elevated significantly (*P*<0.05) as follows: BFBC 20 mg/kg (100%), BCBF 200 mg/kg (678%), GLIB (0.17 mg/kg) (200%) when compared with control normal group. Similarly, 400 mg/kg BCBF causes slight but increased (11%, *P*>0.05) compared with control normal group. Both 200 and 400 mg/kg increased pancreatic catalase activities by 300 and 160%, respectively when compared with control diabetic group.

**Figure 13:**
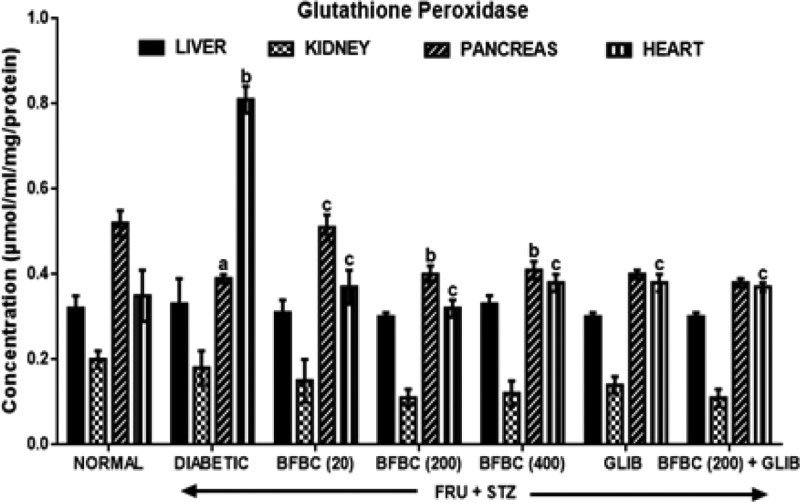
Effects of butanolic fraction of BC on liver, kidney, pancreas, and heart glutathione peroxidase activities of normal and HFSTZ-induced T2D in male Wistar rats Results are represented as mean ± S.E.M.; *n*=6. ^a^*P*<0.05 or ^b^*P*<0.001 when compared with control normal group. ^c^*P*<0.05 when compared with control diabetic group.

Figure 13: Pancreas GPx activity was reduced (*P*<0.05) in the control diabetic rats when compared with the control normal group. However, contrastingly, its activity increased in heart by 131% (*P*<0.001). GLIB did not cause any significant alteration in the GPx levels in the liver, pancreas, and heart, although, renal GPx levels increased by 9 and 14.3% when compared with control groups. Also, the GPx levels following administration of BFBC at the doses administered (20, 200, and 400 mg/kg) were insignificantly different when compared with control groups. Although, a slight increase was obtained at doses 20 mg/kg (31%), 200 mg/kg (2.5%), and 400 mg/kg (5%) in the pancreas when compared with control diabetic group.

**Figure 14:**
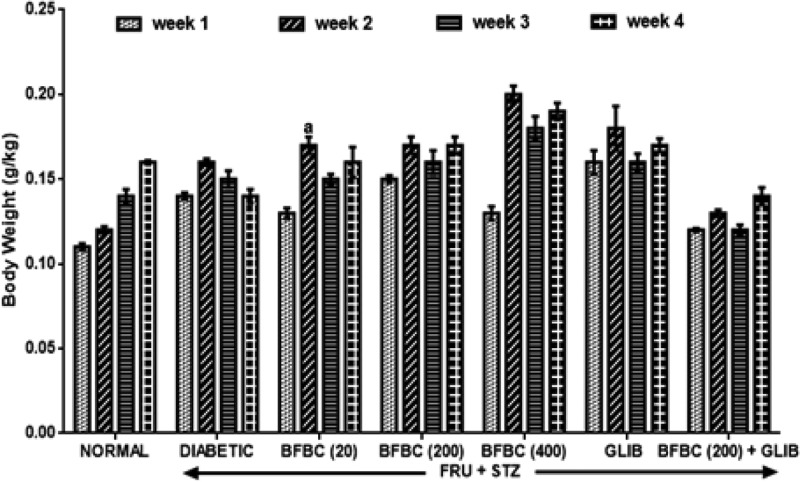
Effects of butanolic fraction of BC on body weight in HFSTZ-induced T2D in male Wistar rats Results are represented as mean ± S.E.M.; *n*=6. ^a^*P*<0.05 when compared with control normal group.

Figure 14: In the second week following fructose administration, there was an increase in body weight in the treated animals. Similarly, a decrease in body weight was obtained during the third week following STZ administration in HFSTZ-induced T2D rats when compared with the control normal group. Treatment of diabetic rats with BFBC (20 mg/kg), BFBC (200 mg/kg), and BFBC (400 mg/kg) further increased the body weights by 21, 21, and 36% when compared with control diabetic rats. Similarly, GLIB treatments increased body weight, although, insignificantly when compared with the control normal group**.**

**Figure 15:**
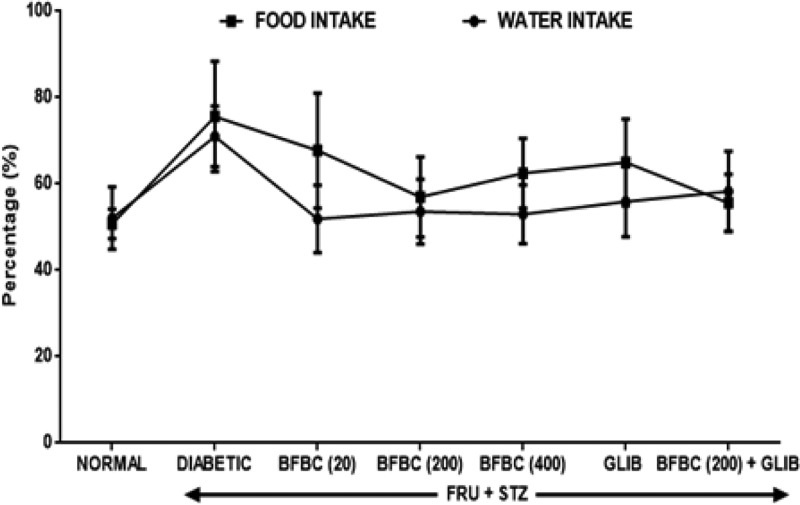
Effects of butanolic fraction of BC on group food and water intake in HFSTZ-induced T2D in male Wistar rats Results are represented as mean ± S.E.M.; *n*=6.

Figure 15: Both increase in food and fluid intake (*P*>0.05) were observed in the diabetic untreated animals when compared with the control normal group. However, treatments with 20, 200, 400 mg/kg, or GLIB improve food and fluid intake in the treated rats when compared with control normal group.

## Discussion

**Figure 16:**
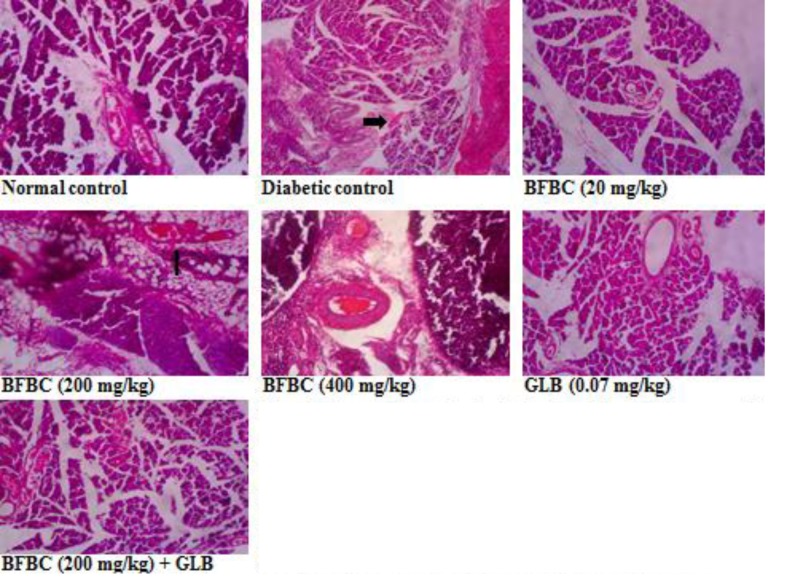
Pancreas section showing the effects of BFBC on the pancreas of normal and T2D male rats Normal control: no visible lesion. Diabetic control: small number of islet of Langerhans and β-cells. BFBC (20 mg/kg): small β-cells with mild inflammation. BFBC (200 mg/kg): islet of Langerhans with moderate inflammation. BFBC (400 mg/kg): moderate inflammation. GLB (0.07 mg/kg): small β-cells with mild inflammation. Abbreviation: BFBC, butanol fraction of BC (H&E stained, Mag. ×400).

**Figure 17:**
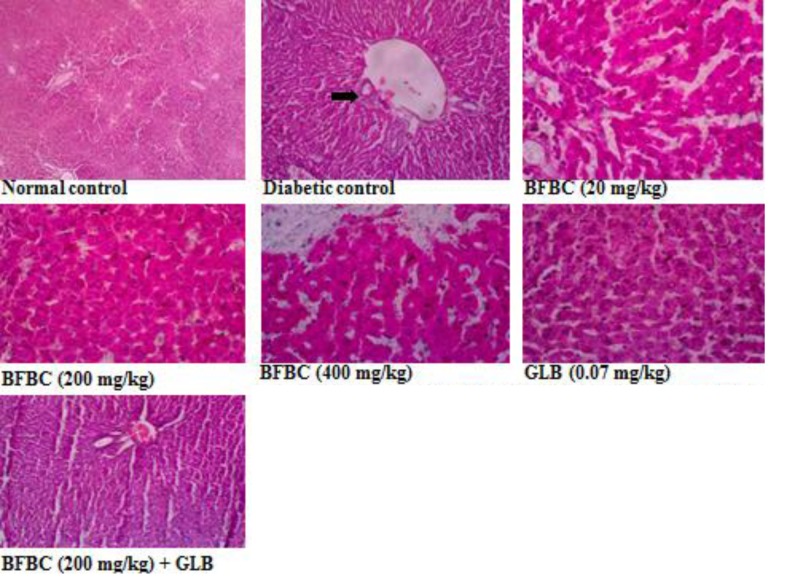
Liver section showing the effects of BFBC on the liver of normal and T2D male rats Normal: no visible lesion. Diabetic: severe centrilobular hepatic necrosis and cellular infiltration. BFBC (20 mg/kg): Moderate cellular infiltration by mononuclear cells. BFBC (200 mg/kg): Mild cellular infiltration by mononuclear cells. BFBC (400 mg/kg): Moderate hepatic inflammation. GLB (0.07 mg/kg): Mild cellular infiltration by mononuclear cells. Abbreviation: BFBC, butanol fraction of BC (H&E stained, ×400).

**Figure 18:**
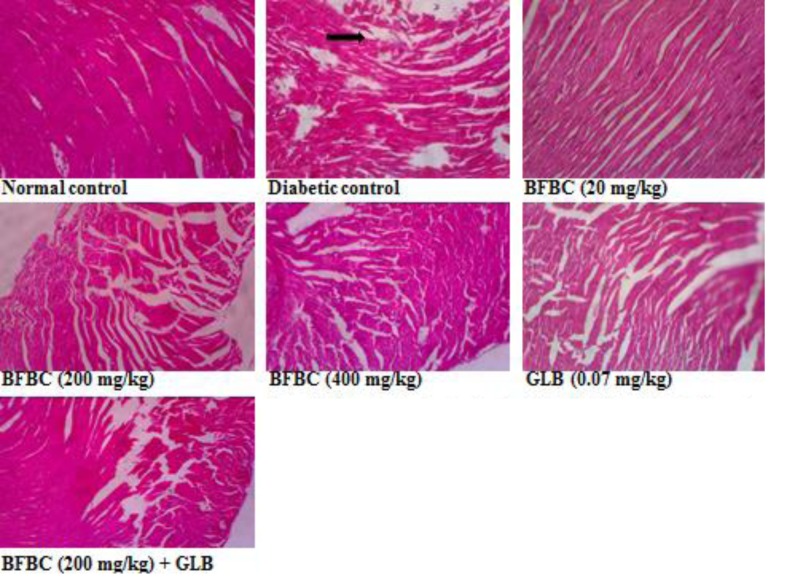
Heart section showing the effects of BFBC on the heart of normal and T2D male rats Normal control: no visible lesion. Diabetic control: large patches of strong fibrous tissue. BFBC (20 mg/kg): large patches of fibrous tissue. BFBC (200 mg/kg): small patches of fibrous tissue. BFBC (400 mg/kg): small patches of fibrous tissue. GLB (0.07 mg/kg): small patches of strong fibrous tissue. Abbreviation: BFBC, butanol fraction of BC (H&E stained, Mag. ×400).

**Figure 19:**
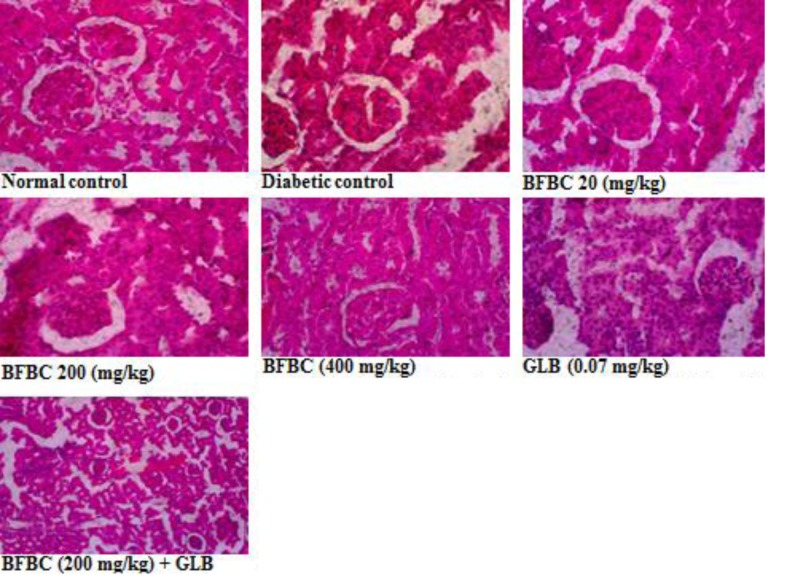
Kidney section showing the effects of BFBC on the kidney of normal and T2D male rats Normal control: no visible lesion, Diabetic control: vascular congestions around the tubules with glomerular atrophy. BFBC (20 mg/kg): vascular congestions with moderate inflammation, BFBC (200 mg/kg): vascular congestions with mild inflammation. BFBC (400 mg/kg): tubular dilations. GLB (0.07 mg/kg): tubular dilations. Abbreviation: BFBC, butanol fraction of BC (H&E stained, Mag. ×400).

**Figure 20:**
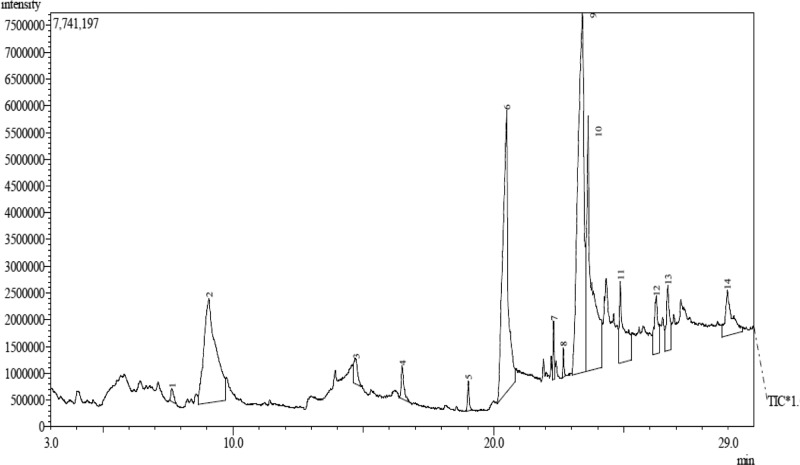
GC-MS chromatogram of butanol fraction of BC seed extract

The use of plants for local and conventional drug discovery and development have thrived for several years. Diabetes is a metabolic disease known to constitute economic problem. However, plants’ derivatives can be used directly or resolved to be used when conventional treatments fail. Although, the notion of relative affordability and effectiveness still remains, low toxicity and minimal side effects have been challenged in recent times [49]. Also, rigorous scientific studies of some herbs and herbal products have been used to update the knowledge for use and toxicological profiles [50,51]. Interestingly, there have been growing interests in therapeutic drug development in tackling T2D, although, not for cure. Several factors including long-term uses and some undesirable side effects which are associated with the currently available therapeutic agents continuing to generate scientific debate. BC seeds have a history of antidiabetic use [52]. However, the main reason for the present study was solely to develop an ideal model for T2D which would closely reflect natural history and metabolic characteristics of human T2D and the possible chemoprotective potential of BC in an experimental animal model. Recent studies have demonstrated the suitability of HFSTZ-induced T2D in rats in order to model the characteristic features obtained in humans [12,13,53–55]. These evidence of its suitability were the major reasons why HFSTZ was employed for use. Thus, the present study investigated the modulatory effects of BFBC seeds extract in HFSTZ-T2D in male Wistar rats. Although, preliminary study on BC shows hypoglycemic activity [22], but, whether this will translate into amelioration of metabolic syndrome of IR, dyslipidemia, and β-cells dysfunction comorbidities which are the hallmarks for the development of T2D and cardiovascular complications have not been evaluated. Also, the implication of insulin during metabolic disorders involving T2D is marked, but, the effect of BC on insulin levels has not been studied. In addition, the present study is the first to characterize the active compounds in BFBC obtained from methanolic seed extracts as well as evaluated both *in vitro* antioxidants and antidiabetic activities. From our results, an oral acute toxicity study using limit dose test of Up and Down Procedure (2001) in male rats show that BFBC may be relatively safe as we observed no mortality following doses of BC methanolic extract up to 4 g/kg. This is a rate-limiting step for a popular demand of several plants species and an important finding that determines the major routes of administration. Rats administered graded doses up to 4 g/kg did not show any visible abnormal behavior traceable to adverse effect of BFBC in terms of restlessness, weakness, fasciculation, dilation of pupils, and increased shallow breathing following 2 h of administration. This is also in agreement with the report of Adisa et al. [22] where no death occurred following a very large amount of BC ethanolic extracts i.p. Application of *in vitro* studies is a step *ab initio* toward pharmacological screening of new compounds. Antioxidants were defined as substances that delay, prevent, or remove oxidative damage to a target molecule [56]. Thus, *in vitro* antioxidant activities of DPPH, NO radical, and HRS activities were assessed for methanol, butanol, hexane, and ethyl acetate fractions of BC methanolic extract *in vitro*, respectively. Amongst the fractions, BFBC has the highest percentage yield of DPPH and HRSA than EFBC which had a better inhibitory activity for NOS (Figure 1B–D). The various ways through which antioxidant activities are manifested have been reviewed [57], but, the emergence of this concept seems to be evolving. α-amylase also constitutes one of the key digestive system enzymes which catalyzes the initial steps during hydrolysis in the body [58]. An inhibition of α-amylase has been suggested to play a key role in the pathogenesis as well as control of diabetes. Our knowledge of modulating post-prandial glucose levels which contribute to treatment of T2D has been updated [59]. However, this attribute is now clinically useful as antidiabetic agents including acarbose, miglitol, voglibose, and a host of others offer therapeutic opportunities. From the results obtained in the present study, BFBC also shows highest α-amylase inhibitory activity (Figure 2). Anti-amylase activity has been confirmed in phenolic and flavonoids containing compounds which have in turn been shown to inhibit this enzyme *in vitro* [58]. The aforementioned were also found to be largely present in BFBC (Figure 1A). Also, untreated diabetic rats show increase in BGLs which provides the suitability for T2D induction, although, blood glucose concentration in normal rats remain unchanged (Figure 3). Similarly, MDA, a metabolite of lipid peroxidation, used to score oxidative stress in diabetic rats was also elevated (Figure 9). This may explains why treatment with HF and STZ may induce T2D through various mechanisms including IR and deterioration in β-cell functions which were observed in these rats. BFBC oral administration in rats however showed time-dependent lowering effects on BGLs, an effect comparable with standard antidiabetic agent, glibenclamide, used in the present study (Figure 3). Our results agree with those of Adisa et al. [22] where hypoglycemic activity of BC seeds in STZ-induced diabetic rats was demonstrated, although, we extended our treatment. BFBC administration showed chemoprotective benefits in diabetic rats and produced comparable actions with BGLs lowering effects of antidiabetic drug used in the present study. There seems to be an enhanced hypoglycemic action between BFBC and glibenclamide combination in treated rats. Further studies are required to know if this action of BFBC will translate into those of sulphurnourea antidiabetics like drugs or others. HFSTZ-induced T2D untreated rats also show sustained serum hyperinsulinemia levels (Figure 7). This may, in part, be linked to a compromised β-cell function which might set a cascade of hyperglycemic effect as against any *in vivo* compensatory action. This is in agreement with Ohly et al. [60] and Srinivasan et al. [61] where low doses of STZ alone or in the present high-fat diet promote symptoms of metabolic syndromes. It appears HFSTZ may utilize this mechanism of exhausting β-cells pancreatic content due to STZ, plus HF-fed rats insulin resistance to catapult susceptibility to diabetogenic effects observed in our study. The subacute administration of BFBC at all doses reversed the abnormally elevated serum insulin in treated animals. More so, since HFSTZ-induced T2D contribute to impaired insulin handling [62]; studies have suggested that low availability of glucose may facilitate immediate lipolysis [63] thereby releasing fatty acid metabolites into the blood circulation [64]. Although, lipid receptors were not reported in our study, but, there were some alterations in levels of serum TC and TG (Figure 5), which may increase the susceptibility to metabolic insults including T2D. In our results, T2D untreated control show abnormally increased LDL levels (Figure 5), whereas, IR following high fructose ingestion in rats is believed to be related to the hypertriglyceridemic effect of fructose [65]. The development of atherosclerosis in T2D has also implicated dyslipidemia as a major culprit [66]. For instance, fructose ingestion sensitizes hepatic production of TG, both by promoting the re-esterification of circulating non-esterified fatty acids and by stimulating *de novo* fatty acid synthesis [67]. Whereas lowering the LDL-cholesterol level may be an important factor in reducing the serum TC level in HFSTZ-induced T2D rats. HDL was elevated when the highest dose of BFBC used was administered to treated rats and when BFBC was combined with glibenclamide. Our study results demonstrated BFBC to be a more potent inhibitor of hypertriglyceridemia than hypercholesterolemia. Since, an abnormal presentation of metabolic disorders is predicated to alter levels of blood lipids that may be due to prolong effects of high fat diet. The aforementioned also shows that HFSTZ-induced T2D may serve as a potential artherogenic substance, although, this may be dose- and time-dependent. Excess water intake and food intake, weight loss, blurred vision, and frequent urination are found in patients with diabetes mellitus [68]. Also, obesity amongst others is a risk factor in most individuals diagnosed with T2D patients [69]. Both increase in nutritional status and water intake (Figure 15) were evident in the HFSTZ-induced T2D rats together with decrease in body weight (Figure 14) when compared with the control animals. Although, we measured neither energy nor urinary output, however, this might be due to the severity of diabetic condition and or energy usage via urinary glucose excretion. Moreover, treatments with doses of BFBC improved body weight when compared with HFSTZ-induced T2D rats. Further, BFBC when administered alone and in diabetic rats did not significantly alter antioxidants biomarker enzymes activities in all rats, although, some modulatory actions were marked (Figures 10–13). Histological assessments showed that sections of the pancreas, liver, kidney, and heart from BFBC-treated animals had reduced tissue damage relative to their controls (Figures 16–19). Whereas, loss of cellular energy due to glucose deficiency has been associated with morphological deterioration in some vital organs [70], studies of energy loss in diabetes animals suggest that effects of intensive insulin therapy on mitochondrial integrity would contribute to the clinical benefits [71]. Although, at this point, it may be impossible to adjudge the degree of injury, but, further molecular studies may help provide tenable conclusions. Also, elevated ALP and AST but not ALT were observed in T2D rats, but were moderately lowered in treated animals (Figure 4). In addition, a perturbed metabolic condition like that observed in T2D patients may drive liver injury or even allow hepatic stellate cells to compromise. Renal function enzyme such as urea level could also be altered which may contribute to modification of treatments in T2D patients. In addition, BFBC alone and in combination with glibenclamide lowered urea and uric acid levels, respectively, in treated rats (Figure 6). We now understand that the process of drug development depends on the identifiable compounds presence. Thus, a detailed biological assay is required before dosage formulations followed by clinical studies are established. Results obtained from GC-MS analysis (Figure 20) revealed 14 bioactive compounds in BFBC (Table 3), although, only half were found to be present in large amounts. These include oleic acid, stearic acid, 2-methyl-pyrrolidine-2-carboxylic acid, n-hexadecanoic acid, 13-docosenoic acid, l-[-]-4-hydroxy-1-methylproline, and 2,3-dihydro-3,5-dihydroxy-6-methyl-4H pyran-4-one. Thus, some derivatives of BFBC may be useful in combating the prevention and treatment of problems associated with T2D or even act possibly as an inhibitor of ambiental factor on genetic T2D. For instance, previous reports have indicated oleic acid for antidiabetic [72] and hypocholesterolemic [73]; steric acid for antidiabetic [74] and hypocholesterolemic [75]; 2-methyl-pyrrolidine-2-carboxylic acid for antidiabetic [76] and anticancer [77]; n-hexadecanoic acid for antioxidant [78], hypocholesterolemic [78]; 13-docosenoic acid for antioxidant [79] and 3-dihydro-3,5-dihydroxy-6-methyl-4H pyran-4-one for antioxidant [80], respectively. Also, purification of each bioactive compound is essential in order to increase drug development. Antioxidant levels were modulated by BFBC treatments in T2D animals. Although, our study demonstrates the efficacy of BFBC in T2D in rats; however, implications are that modulation of neutrophil and lymphocyte levels were found in T2D rats treated with BFBC. Whether this later will translate into an adverse herb reaction requires holistic toxicological investigation.

## Conclusion

In conclusion, the present study further confirms the current model that combines high fructose and STZ to achieve impaired glucose tolerance causing glycemic imbalance which bears a resemblance to the metabolic characteristics of T2D in humans. Also, it was suitable to produce *in vivo* oxidative stress, hyperinsulinemia, dyslipidemia as well as deterioration in β-cell function in T2D rat experiment. However, BFBC doses used in the present study demonstrated *in vitro* and *in vivo* antioxidants, antihyperglycemic, antihyperlipidemic activities against HFSTZ-induced T2D rats. Also, the results obtained from the present study show possible chemoprotective potentials similar to glibenclamide, although, it shows tendency to modulate hematological indices and renal function. The most abundant bioactive compounds present in BFBC are oleic, stearic, 2-methyl-pyrrolidine-2-carboxylic, and n-hexadecanoic acid. Further, some derivatives of BFBC may be useful in the prevention and treatment of problems associated with T2D, hence the folklore medicine use. Studies to show chronic toxicity and unravel the mechanisms of antidiabetic actions are essential.

## Abbreviations

ALP: alkaline phosphatase
ALT: alanine aminotransferase
AST: aspartate aminotransferase
BC: *Buchholzia coriacea*
BFBC: butanol fraction BC
CMC: carboxymethyl cellulose
DPPH: 2,2-diphenyl-1-picrylhydrazine
EFBC: ethyl acetate fraction BC
FBGL: fasting blood glucose level
FF: fructose feeding
GLIB: glibenclamide
GPx: glutathione peroxidase
HDL: high-density lipoprotein
HFBC: hexane fraction BC
HFSTZ: high-fructose fed streptozotocin
HRS: hydroxyl radical scavenging
H&E: Hematoxylin and Eosin
IR: insulin resistance
i.p.: intraperitoneal
LDL: low density lipoprotein
LSD: least square difference
MDA: malondialdehyde
NIST: national institute science and technology
NOS: nitric oxide scavenging
OECD: organisation for economic cooperation and development
PCV: packed celll volume
SOD: superoxide dismutase
STZ: streptozotocin
TBARS: Thiobarbituric acid reactive substance
TC: total cholesterol
TG: triglyceride
T2D: type 2 diabetes
T2DM: T2D mellitus
